# Effects of a preoperative forced-air warming system for patients undergoing video-assisted thoracic surgery: A randomized controlled trial

**DOI:** 10.1097/MD.0000000000023424

**Published:** 2020-11-25

**Authors:** Yan Xiao, Rui Zhang, Na Lv, Chunmiao Hou, Chunguang Ren, Huiying Xu

**Affiliations:** a Department of Operation Room; b Department of Anaesthesiology, Liaocheng People's Hospital, Liaocheng, Shandong, China.

**Keywords:** anesthesia, erector spinous nerve block, perioperative inadvertent hypothermia, preoperative warming

## Abstract

**Background:**

The incidence of intraoperative hypothermia is still high despite the proposal of different preventive measures during thoracoscopic surgery. This randomized control study evaluated the effects of 30-minute prewarming combined with a forced-air warming system during surgery to prevent intraoperative hypothermia in patients undergoing video-assisted thoracic surgery under general anesthesia combined with erector spinae nerve block.

**Methods:**

Ninety-eight patients were randomly and equally allocated to prewarming or warming groups (n = 49 each). The primary outcome was the incidence of intraoperative hypothermia. Secondary outcomes were core temperature, irrigation and infused fluid, estimated blood loss, urine output, type of surgery, intraoperative anesthetic dosage, hemodynamics, recovery time, the incidence of postoperative shivering, thermal comfort, postoperative sufentanil consumption and pain intensity, patient satisfaction, and adverse events.

**Results:**

The incidence of intraoperative hypothermia was significantly lower in the prewarming group than the warming group (12.24% vs 32.65%, *P* *=* .015). Core temperature showed the highest decrease 30 minutes after surgery start in both groups; however, the rate was lower in the prewarming than in the warming group (0.31 ± 0.04°C vs 0.42 ± 0.06°C, *P* *<* .05). Compared with the warming group, higher core temperatures were recorded for patients in the prewarming group from T1 to T6 (*P* *<* .05). Significantly fewer patients with mild hypothermia were in the prewarming group (5 vs 13, *P* *=* .037) and recovery time was significantly reduced in the prewarming group (*P* *<* .05). Although the incidence of postoperative shivering was lower in the prewarming group, it was not statistically significant (6.12% vs 18.37%, *P* *=* .064). Likewise, the shivering severity was similar for both groups. Thermal comfort was significantly increased in the prewarming group, although patient satisfaction was comparable between the 2 groups (*P* *>* .05). No adverse events occurred associated with the forced-air warming system. Both groups shared similar baseline demographics, type of surgery, total irrigation fluid, total infused fluid, estimated blood loss, urine output, intraoperative anesthetic dosage, hemodynamics, duration of anesthesia and operation time, postoperative sufentanil consumption, and pain intensity.

**Conclusion:**

In patients undergoing video-assisted thoracic surgery, prewarming for 30 minutes before the induction of anesthesia combined with a forced-air warming system may improve perioperative core temperature and the thermal comfort, although the incidence of postoperative shivering and severity did not improve.

## 1 Introduction

Video-assisted thoracic surgery (VATS) has become more and more widespread for its faster post-operative recovery rate over the past two decades.^[1]^ Recently, ultrasound-guided erector spinae plane block (ESPB) combined with general anesthesia has been used as a new multimodal analgesia regimen for VATS.^[2]^ However, both anesthesia methods are able to inhibit the thermoregulatory mechanisms of patients and result in hypothermia if a suitable warming strategy is not taken.^[3]^ Inadvertent perioperative hypothermia (IPH), defined as perioperative core temperature of <36.0°C, has been considered one of the most common complications for patients undergoing VATS. A previous study reported that the incidence of IPH was as high as 50%, which depended on various factors such as a cold operating room environment, duration and type of surgical procedure, anesthetic technique, patient demographics, and positioning.^[4,5]^ Perioperative hypothermia can decrease the metabolic rate and cardiac output, prolong drug metabolism, increase the incidence of postoperative infection and shivering, delay surgical wound healing, alter clotting functions, and impair immune function. Finally, the length of hospital stay and health costs are reported to increase.^[6,7]^

The core body temperature of the majority of patients may decrease by 0.5°C to 1.0°C within the first hour following induction of anesthesia or peripheral nerve block because of the redistribution of body temperature, which is determined by the intensity of vasodilation, radiation (the most common one) and convection by environmental temperature, and the duration of the exposure to the environment.^[8]^ Several methods and devices, such as the use of fluid warmers, resistive heating, convective and conductive devices, have been adapted to actively warm patients; however, their relative effectiveness is still controversial.^[9,10]^

Preoperative warming as a preventive strategy to control the surgical patient's thermal management has been strongly recommended by recent German guidelines.^[11]^ It can increase the peripheral tissue temperature and reduce the central-to-peripheral temperature gradient, prevent thermal redistribution before induction of anesthesia, and it ultimately reduces the overall incidence of hypothermia.^[12]^ The most commonly used device for preoperative warming is a forced-air warming system, which has been strongly recommended by the National Institute for Health and Care Excellence, especially for patients at high risk of IPH and for surgeries lasting more than 30 minutes.^[13]^ However, a preoperative active warming method has been used in only 20% of the patients according to a previous study.^[12]^ Furthermore, the method is inadequate for some types of surgery because of the insufficient rewarming time available, although intraoperative forced-air warming can eventually restore normothermia.^[14]^ The aim of this study was to investigate the effects of prewarming for 30 minutes combined with a forced-air warming system on intraoperative hypothermia in patients undergoing VATS under general anesthesia combined with ESPB.

## 2 Material and methods

### 2.1 Patients

This trial was approved by the Institutional Review Board of our hospital and was registered at chictr.org (ChiCTR-IPR-15007229). Written informed consent was obtained from all patients. The inclusion criteria were: patients aged 45 to 60 years with American Society of Anesthesiology (ASA) grades I to II; operation time between 1 hour and 3 hours; who underwent elective VATS under general anesthesia combined with ESPB between December 2016 and June 2019. Exclusion criteria were: patients with endocrine disorders (eg, thyroid disease, dysautonomia, Cushing syndrome); patients with peripheral vascular disease (eg, Raynaud syndrome), impaired respiratory function (eg, chronic obstructive pulmonary disease, asthma) or vascular disease (eg, coronary artery disease with New York Heart Association >II); febrile patients (>37.3°C) or tympanic temperature <36.0°C; delay time (from the end of prewarming to the start of intraoperative forced-air warming) longer than 10 minutes; body mass index (BMI) >30 kg/m^2^.

### 2.2 Randomization and blinding

A computer-generated randomization table was used for participant allocation. On the day before surgery, one nurse who was unaware of the study details performed the preoperative evaluation and educated patients on how to use the patient-controlled intravenous analgesia pump. Another nurse, also unaware of the details of the study, opened the sealed envelope and randomly allocated the patient to the prewarming group (n = 49) or warming group (n = 49) when the patient entered the operating room. The anesthesiologist and surgeon were all blinded to the study conditions.

### 2.3 Ultrasound-guided ESPB

None of the patients received premedication before surgery. Patients were under standardized monitoring with 5 L/min oxygen before they being placed in a lateral position in the anesthesia preparation room. ESPB was performed as described in the previous study by the same anesthesiologist.^[15]^ The probe was placed 2 to 3 cm lateral to the T5 transverse process longitudinally. After visualizing the trapezius, rhomboid major, erector spinae muscles and the transverse processes, an 8-cm, 22-gauge needle was inserted in the cephalad-to-caudad direction with a shallow trajectory in the fascial plane, deep to the erector spinae muscle with an in-plane approach. A volume 2 mL saline was injected to confirm the proper injection site and then a volume of 30 mL 0.33% ropivacaine were injected.

### 2.4 Intraoperative anesthesia management

Anesthesia was induced using 1.5 to 2.5 mg/kg propofol, 0.2 μg/kg sufentanil, 0.2 mg/kg cisatracurium, and 1 mg/kg lidocaine. The position of double lumen tube was verified using fiberoptic bronchoscopy. 0.1 mg/kg dexamethasone was administered for prophylaxis of postoperative nausea and vomiting before surgical incision.^[16]^ Dosages of 2 to 4 μg/mL propofol with target controll infusion, 0.2 to 0.7 μg/kg/h dexmedetomidine, and 0.1 to 0.2 μg/kg/min remifentanil were adjusted to target a bispectral index between 40 and 60 during surgery. Cisatracurium (0.1 mg/kg) was infused as necessary to maintain muscle relaxation. One-lung mechanical ventilation was set with a tidal volume of 4 to 6 mL/kg and a peak airway pressure of <25 cm H_2_O according to protective lung ventilation strategy.^[17]^ A dosage of 5 mg intravenous tropisetron was given about 30 minutes before the end of surgery for postoperative nausea and vomiting prophylaxis. Neuromuscular blockade was antagonized by 0.01 mg/kg atropine and 0.02 mg/kg neostigmine at the end of surgery. All VATS procedures were performed by the same surgeon through three incisions without carbon dioxide insufflations.^[18]^ Patient-controlled intravenous analgesia was programmed to deliver 0.02 μg/kg/h sufentanil and 0.02 μg/kg sufentanil bolus, followed by a 5-minute lockout period with 1 hour limit 0.16 μg/kg sufentanil. A rescue dose of 30 mg ketorolac was given if visual analogue scale at rest scored >3 or in accordance with the patients’ demands.

### 2.5 Perioperative warming management

The temperature of the operation room was adjusted to 22.0 ± 1.0°C, with relative humidity ranging 40% to 60% according to the National Institute for Health and Care Excellence Clinical Guidelines.^[19]^ In the prewarming group, patients were prewarmed for 30 minutes after ESPB in the anesthesia preparation room using a full body forced-air warming blanket (Model 750, 3M Bair Hugger, USA) set to 38.0°C and then with an upper body forced-air warming blanket set to 38.0°C during surgery. In the warming group, patients were warmed with upper body forced-air warming blanket set to 38°C as soon as they entered the operating room. The disposable upper body blanket was positioned in accordance with a previous study.^[5]^ After surgery, all patients were extubated and transferred to the post-anesthesia care unit (PACU) with the full body forced-air warming blanket until they left the PACU. The warming system was paused if the core temperature of the patients was >37.5°C to 43.0°C, or if core temperature was <36.0°C.

All patients received intravenous and irrigation fluid warming set to 38.0°C. Body core temperatures were recorded using 2 different thermometers. The temperature of patients was measured with infrared tympanic thermometer before the induction of anesthesia and after surgery for less intrusion and ease of operation. An esophageal probe was immediately placed in the upper esophagus near the nasopharynx after induction of anesthesia and the continuous monitorization was recorded.

### 2.6 Outcomes

The primary outcome was the incidence of intraoperative hypothermia. Secondary outcomes were core temperature, total irrigation fluid, total infused fluid, estimated blood loss, urine output, type of surgery, intraoperative anesthetic dosage, hemodynamics, recovery time (from patients arriving at the PACU to Steward >4), the incidence of postoperative shivering, thermal comfort, postoperative sufentanil consumption, pain intensity (recorded at 1, 4, 8, 12, 24, and 48 hours postoperatively), patient satisfaction, and adverse events.

Core temperature was recorded at the following time points: arrival at the operating room (T0); leaving the anesthesia preparation room (T1); before anesthesia induction (T2); before incision (T3): at 10 minutes (T4), 20 minutes (T5), 30 minutes (T6), and 60 minutes (T7) after the onset of the operation; at the end of the operation (T8); on arrival at the PACU (T9); 5 minutes (T10), 10 minutes (T11), and 15 minutes (T12) after arriving at the PACU; and on leaving the PACU (T13). The severity of hypothermia was graded based on the core temperature as follows: mild hypothermia (35.5°C–35.9°C), moderate hypothermia (35.0°C–35.4°C), and severe hypothermia (<35.0°C). Shivering was scored using a visual scale as follows: 0, no visible or palpable shivering; 1, palpable and visible shivering or noise on the electrocardiogram; 2, visible shivering of the face and neck; 3, visible shivering of the chest or torso; and 4, generalized shivering with or without chattering teeth.^[20]^ Thermal comfort was measured using an 11-point Likert scale: 0 = entirely cold, 10 = fully hot. Pain intensity was scored on an 11-point VAS (0, no pain; 10, worst pain). Satisfaction of patients was assessed using an 11-point Likert scale: 0 = entirely unsatisfied, 10 = fully satisfied.

### 2.7 Statistical analysis

Based on our pilot study, 32.4% of patients experienced intraoperative hypothermia in the warming group, assuming a difference of 15% between the 2 groups as clinically significant. A sample size of 42 patients per group (α = 0.05, β = 0.8; PASS 11.0, NCSS Statistical Software, Kaysville, Utah) and considering a dropout rate of 15%, the study population was set at 98, with 49 patients per group.

The Kolmogorov–Smirnov test was used to assess distribution of variables. Homogeneity of variance was determined using Levene tests. Normally distributed data were expressed as mean standard deviation, non-normally distributed data were expressed using median (interquartile range), and categorical data was expressed as number (n) and percentage (%). Inter-group comparisons were performed using repeated-measures analysis of variance. Bonferroni correction was used for post-hoc multiple comparisons. Non-normally distributed data were analyzed with the Kruskal–Wallis test, chi-squared tests, or Fisher exact tests. Probability (*P*) values <.05 were considered statistically significant. Statistical analysis was performed with SPSS version 22.0 (SPSS Inc., Chicago, IL).

## 3 Results

### 3.1 Baseline characteristics

**Figure 1:**
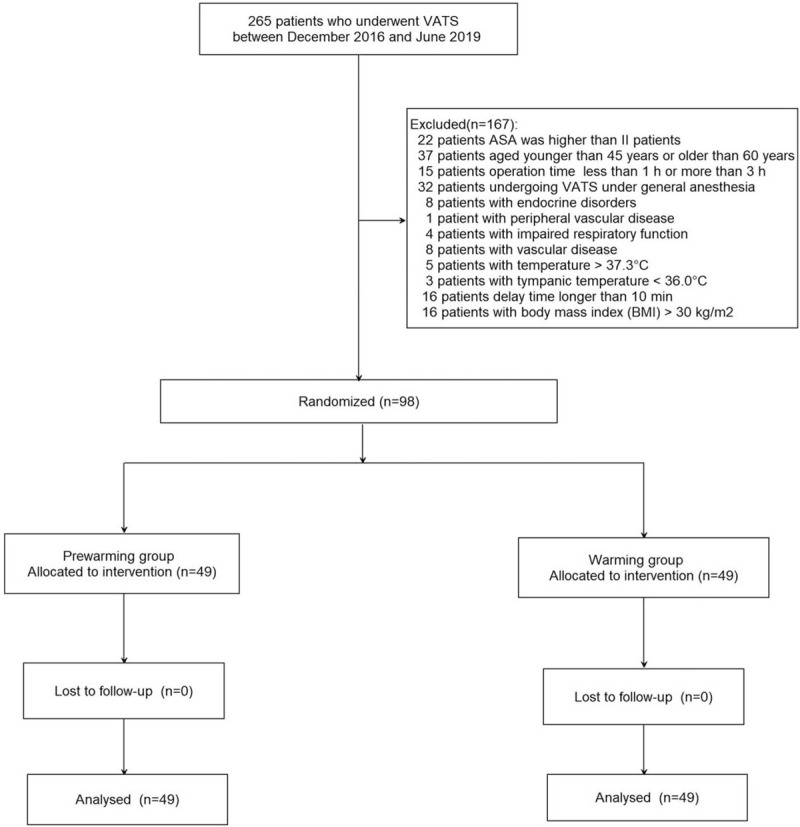
Flow diagram of patient enrollment.

Figure 1 shows the flow diagram of patient enrollment in this study. A total of 265 patients who underwent VATS between December 2016 and June 2019 were recruited. Of these, 167 patients were excluded for the following reasons: 22 patients’ ASA was higher than grade II, 37 patients aged <45 years or >60 years, 15 patients with operation time <1 hour or >3 hours, 32 patients subjected to VATS under general anesthesia, 8 patients with endocrine disorders, 1 patient with peripheral vascular disease, 4 patients with impaired respiratory function, 8 patients with vascular disease, 5 patients with temperature >37.3°C, 3 patients with tympanic temperature <36.0°C, 16 patients with delay time >10 minutes, 16 patients with BMI >30 kg/m^2^. Finally, 98 patients were randomly allocated to either the prewarming (n = 49) or warming groups (n = 49).

There were no significant differences between the 2 groups with respect to age, BMI, ASA grade, sex, body surface area, operating room temperature, pulmonary function or comorbidities (*P >* .05, Table 1).

**Table 1:** Patients’ baseline characteristics in the 2 groups.

	Prewarming group (n = 49)	Warming group (n = 49)	*P*-values
Age (yrs)	53.81 ± 7.26	56.50 ± 6.71	.057
BMI (kg/m^2^)	22.04 ± 1.21	21.86 ± 1.17	.454
Sex (female/male, n)	16/33	23/26	.149
Body surface area (m^2^)	1.79 ± 0.21	1.76 ± 0.22	.490
ASA I/II (n)	12/37	9/40	.460
OR temperature (°C)	21.95 ± 0.52	22.05 ± 0.32	.252
Location (left/right, n)	32/17	38/11	.180
FEV1/FVC (%)	93.27 ± 4.05	90.58 ± 3.61	.071
Comorbidity, n (%)			.922
Hypertension	15 (30.61%)	17 (34.69%)	
Diabetes mellitus	4 (8.16%)	6 (12.24%)	
Coronary heart disease	3 (6.12%)	5 (10.20%)	

### 3.2 Perioperative data

**Figure 2:**
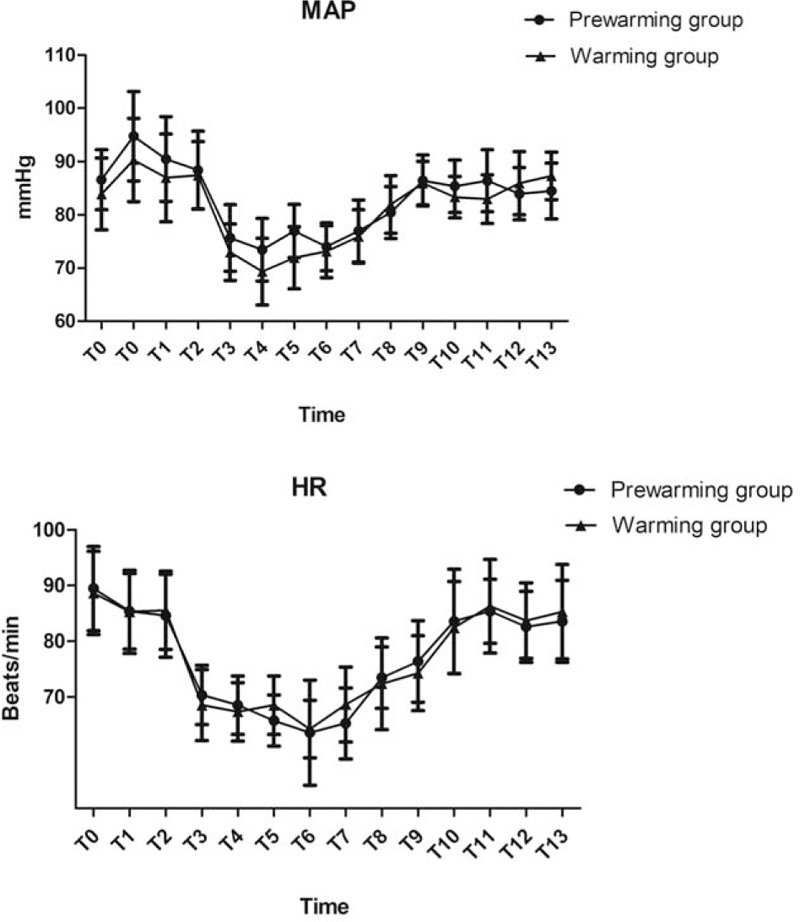
Hemodynamics between the 2 groups.

There was no significant difference between the 2 groups with respect to type of surgery, total irrigation fluid, total infused fluid, estimated blood loss, urine output, intraoperative anesthetic dosage, duration of anesthesia or operation (*P* *>* .05, Table 2). However, recovery time was significantly reduced in the prewarming group (*P* *<* .05, Table 2). Hemodynamics were also comparable between the 2 groups (*P* *>* .05, Fig. 2).

**Table 2:** Intraoperative data in the 2 groups.

	Prewarming group (n = 49)	Warming group (n = 49)	*P*-values
Total irrigation fluid (ml)	328.27 (210.51–583.23)	402.14 (235.19–623.51)	.351
Total infused fluid (ml)	783.15 (562.89–1283.56)	692.02 (393.49–1029.45)	.187
Estimated blood loss (ml)	154.27 (76.39–209.24)	140.78 (98.38–239.19)	.525
Urine output (ml)	483.83 (292.39–893.23)	540.38 (284.77–982.39)	.203
Duration of surgery (min)	126.62 ± 24.09	135.38 ± 28.71	.102
Duration of anesthesia (min)	148.37 ± 20.71	153.23 ± 24.22	.286
Type of surgery, n (%)			.743
Wedge resection	6 (12.24%)	9 (18.37%)	
Segmentectomy	8 (16.33%)	10 (20.41%)	
Lobectomy	32 (65.31%)	28 (57.14%)	
Mediastinal tumor excision	3 (6.12%)	2 (4.08%)	
Dexmedetomidine (μg)	54.09 ± 6.83	59.66 ± 4.81	.095
Propofol (mg)	942.91 (842.86–1288.29)	989.28 (874.37–1391.83)	.729
Remifentanil (mg)	0.55 ± 0.11	0.60 ± 0.06	.128
Cisatracurium (mg)	19.35 ± 4.82	20.15 ± 5.73	.455
Sufentanil (μg)	17.12 ± 2.01	16.87 ± 1.78	.515
Recovery time (min)	17.37 ± 4.25	26.73 ± 6.39^∗^	.001

**Figure 3:**
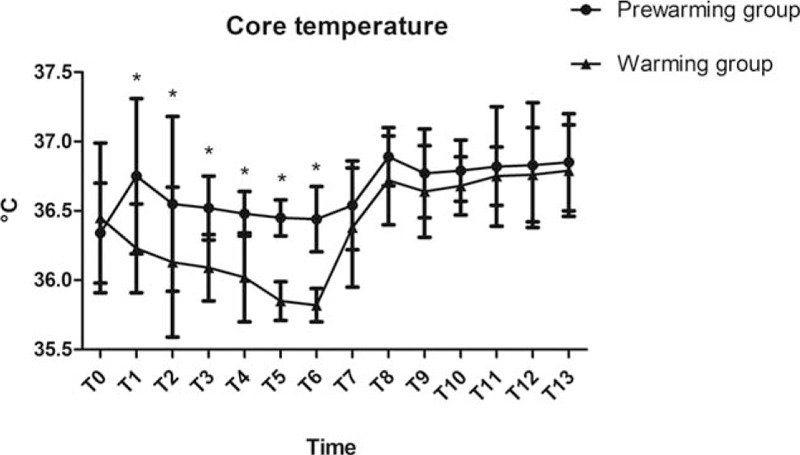
Core temperature between the 2 groups. Core temperature was recorded at the following time points: arrival at the operating room (T0), leaving the anesthesia preparation room (T1), before anesthesia induction (T2), before incision (T3), 10 minutes (T4), 20 minutes (T5), 30 minutes (T6), 60 minutes (T7) after the onset of operation, at the end of operation (T8), arriving at the PACU (T9), 5 minutes (T10), 10 minutes (T11), 15 minutes (T12) after arriving at the PACU and leaving PACU (T13). ^∗^*P* *<* .05 vs Warming group. PACU = post anesthesia care unit.

**Figure 4:**
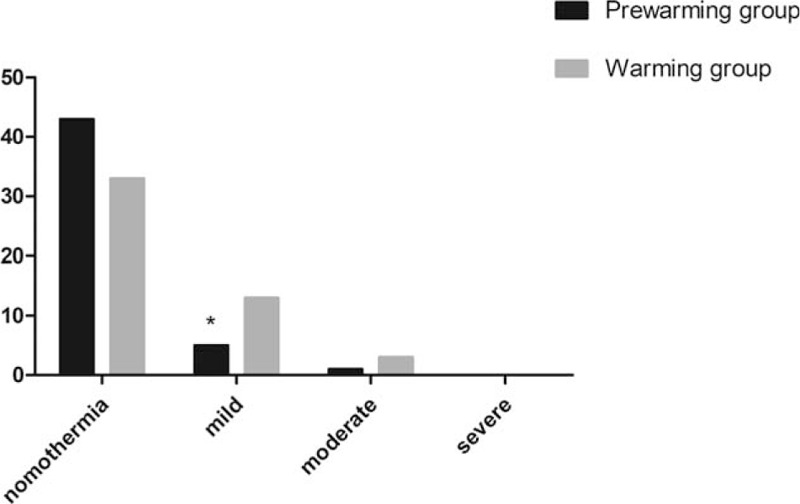
Incidence and severity of hypothermia between the 2 groups. ^∗^*P* *<* .05 vs Warming group.

**Figure 5:**
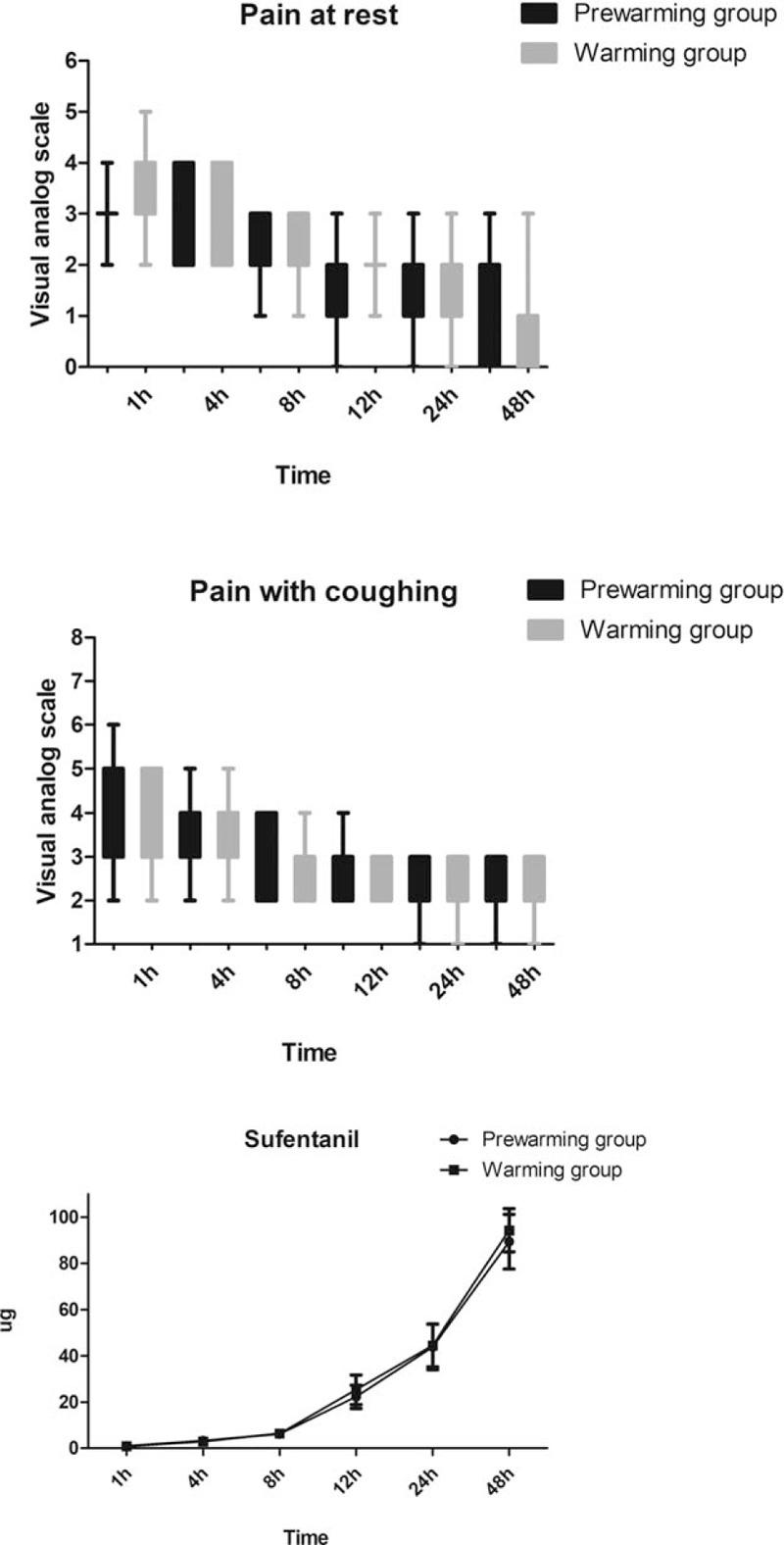
Postoperative sufentanil consumption and pain intensity during the first 48 hours after surgery between the 2 groups.

The core temperature had the highest decrease in both groups during half an hour after the onset of the procedure. However, the rate was lower in the prewarming group than that in the warming group (0.31 ± 0.04°C vs 0.42 ± 0.06°C, *P* *<* .05, Fig. 3). Compared with the warming group, patients with higher core temperature were recorded in the prewarming group from T1 to T6 (*P* *<* .05, Fig. 3). The incidence of intraoperative hypothermia was significantly reduced in the prewarming group (12.24% vs 32.65%, *P* = .015). Only in patients with mild hypothermia was incidence significantly reduced in the prewarming group (5 vs 13, *P* *=* .037, Fig. 4). Patients with moderate hypothermia was comparable between the 2 groups (1 vs 3, *P* *=* .307, Fig. 4). None of the patients experiencing severe hypothermia in the 2 groups showed improvement. There was no difference with respect to postoperative sufentanil consumption and pain intensity during the first 48 hours after surgery (*P* *>* .05, Fig. 5).

Though the incidence of postoperative shivering was lower in the prewarming group, the difference was not statistically significant (6.12% vs 18.37%, *P* *=* .064). At the same time, there was no significant difference in the shivering severity between the 2 groups (*P* *>* .05, Table 3). Though the thermal comfort was significantly increased in the prewarming group, satisfaction of patients was comparable between the 2 groups (*P* *>* .05, Table 4). We also did not record any adverse events associate with the forced-air warming system.

**Table 3:** Comparison of the shivering grade between the 2 groups.

	Prewarming group (n = 49)	Warming group (n = 49)	*P*-values
Grade 0	46 (93.88%)	40 (81.63%)	.064
Grade 1	2 (4.08%)	7 (14.29%)	.080
Grade 2	1 (2.04%)	2 (4.08%)	1.000
Grade 3	0	0	1.000
Grade 4	0	0	1.000

**Table 4:** Patients’ thermal comfort and atisfaction between the two groups.

	Prewarming group (n = 49)	Warming group (n = 49)	*P*-values
Thermal comfort	8.78 (7.89–9.34)	7.83 (6.88–9.03)^∗^	.034
Satisfaction	8.28 (7.46–9.45)	7.76 (7.34–9.03)	.317

## 4 Discussion

This randomized controlled study showed that for patients undergoing VATS, a 30-minute prewarming period before the induction of anesthesia combined with a forced-air warming system could improve perioperative core temperature and the patient's thermal comfort, albeit with no improvement in postoperative shivering or severity.

Patients undergoing VATS are at high risk of hypothermia because a large portion of the pleural surface is exposed to ambient cold air causing substantial evaporative heat loss compared to extensive abdominal surgery.^[21]^ Previous retrospective studies have reported that the incidence of postoperative hypothermia was more than 50% during VATS.^[22]^ However, this incidence may be underestimated. A survey in 17 European countries revealed that fewer than 40% of patients under general anesthesia were actively warmed and less than 20% of patients were monitored by body temperature.^[23]^ In contrast with this conclusion, over 80% of patients under general anesthesia were actively warmed and the core temperature of nearly all patients was monitored from when they entered the operating room up to discharge from the PACU in our operating room. A forced-air body warming system is one of the most commonly used warming devices in clinical use because of its convenience, effectiveness, and low cost. Recently, a previous study reported that an upper body forced-air warming blanket far outweighs the benefit of a lower body forced-air warming blanket to prevent hypothermia during thoracoscopic surgery in the lateral decubitus position.^[5]^ As a result, we adopted a heating strategy using an upper body forced-air warming blanket in both the experimental groups even though the efficiency of warming by this type of blanket also has its limitations. Considering the operating room efficiency and the results of a previous study, patients in the prewarming group were prewarmed for 30 minutes before the induction of anesthesia using full body forced-air warming system set to 38.0°C.^[24]^

In our study, patients had a higher basal core temperature, which may be due to none of the patients received premedication before surgery. A previous study showed that benzodiazepines could influence the balance between heat production and cutaneous heat loss, and produce a concentration-dependent decrease in core temperature by 0.3°C to 0.6°C, which impaired tonic thermoregulatory vasoconstriction.^[25]^ Likewise, patients with a tympanic temperature of less than 36.0°C were excluded from the study, even though a core temperature <36.0°C could be considered as normal in a subset of normal individuals.^[26]^ Consistent with previous studies, patients in the prewarming group experienced a smaller drop in core temperature after induction of anesthesia in our study. The reason may be due to the redistribution of heat from the core to the periphery rather than from heat loss from the body. The strategy of prewarming can increase the patients’ heat content, produce higher skin temperatures, and reduce the resulting core-to-peripheral temperature redistribution.^[4,27,28]^ A previous study investigating only abdominal surgeries reported that co-warming was as effective as prewarming in preventing intraoperative hypothermia.^[29]^ The possibility of temperature loss was mainly due to intraoperative hypothermia which was minimized by the continued intraoperative warming in their study.^[29]^ However, the most reasonable explanation is that hypothermia can only be effectively treated by intraoperative active warming after a core-to-peripheral temperature redistribution phase.^[30]^

Heat stored in the peripheral compartment is gradually lost to the cold environment once active prewarming is stopped. As a result, the potential benefits of preoperative forced-air warming are transient. There is also evidence of the likelihood of a core temperature <36.0°C increasing by 4.9%, with a one minute of delay of initiating intraoperative forced-air warming.^[31]^ Taking all these factors into consideration, we only included patients with a delay time (from the end of prewarming to the start of the intraoperative forced-air warming) of less than 10 minutes in our study. As a result, the highest decrease in core temperature in both groups occurred during the first half hour after the onset of the surgical procedure and not during the period between the preoperative holding and induction of anesthesia as reported by a previous study.^[32]^ Furthermore, we also observed a mild decrease in the core temperature during the period from the end of the surgery to the arrival at the PACU in both groups. The reason may be due to the temporary interruption in active warming throughout the transfer process and the not fully metabolized anesthetics used during the procedure.^[33]^

The core temperature was also higher than previous studies partly because of the age difference of the recruited patients. According to the multiple linear regression analysis of a previous study, the significant decrease in core temperature was associated with advanced age, which was especially true for elderly patients with impaired vasoconstriction, more likely shivering response to hypothermia, reduced subcutaneous fat layer, and frail constitution.^[4,34]^ Further, because of the lower basal metabolic rate, the body temperature of females may be lower than that of males.^[35]^ However, the difference in the sex ratio between the 2 groups was not statistically significant in our study.

Previous studies also reported that higher BMI correlated with lower perioperative hypothermia, whereby a higher BMI was shown to strongly correlate with the estimated body fat percentage, which is associated with lower thermal conductivity, higher leptin levels, higher metabolic rate and body heat, and less heat redistribution from the core to peripheral tissues after anesthetic induction.^[36–38]^ The BMI of our patients were similar and were less than 30 kg/m^2^ across both groups, which was relatively lower compared with a previous study.^[37]^ The incidence of intraoperative hypothermia reduced by 20% (12.24% vs 32.65%) in our study, which was similar to the results of patients undergoing total hip arthroplasty.^[32]^ The reason may be partly due to the addition of preoperative ESPB to the protocol, which may have reduced the total amount of anesthetics administered in patients undergoing VATS. Although ESPB could attenuate thermosensors and afferent neural pathways, which have been proven to play a crucial role in the regulation of thermoregulatory behavior, this impact may be smaller than intraspinal anesthesia given both the lower concentration of anesthetics administered and the longer onset time.^[39]^ Therefore, it appears that ESPB combined with general anesthesia may be beneficial for perioperative temperature management for patients undergoing VATS.^[40]^

Although a previous study confirmed that most anesthetics lower the threshold for shivering,^[41]^ there was no significant difference with respect to the incidence of shivering and its severity in our study. We adopted the warmed intravenous and irrigation fluids because 1 L of unheated crystalloid could reduce the core temperature by 0.25°C to 0.30°C and this was found to be effective in reducing shivering in recent study.^[5]^ Consistent with this result, the incidence of postoperative shivering in our trial was lower than a previous study not using warmed fluids during surgery.^[42]^ Furthermore, the lower consumption of opioids may also contribute to the lower incidence of shivering as there is a lower level of catecholamines resulting from pain and anxiety.^[43]^

We did not record any differences with respect to the amount of bleeding between the two groups. However, the duration of perioperative hypothermia may be associated with increased intraoperative blood loss and the relative risk for transfusion, as reported in a recent retrospective analysis with 50,000 patients of the Cleveland Clinic.^[44]^ The reason may be due to the negative effect on platelet function, reduced the concentrations of various coagulation factors and fibrinogen, which inhibit the enzymes of the coagulation cascade and the activation of the blood fibrinolysis system.^[45,46]^ This difference may be related to both the type of surgery and the sample size of patients in the 2 reported studies. The satisfaction of patients in the prewarming group was higher partly because of the improved patients’ thermal comfort. However, the difference was not statistically significant. Consistent with previous study, we also did not record any adverse events associated with the forced-air warming system.^[47]^

This study has the following limitations: First, we did not record the incidence of infection in this trial as previous study reported that convective warming devices may potentially lead to surgical site infection due to the disruption of unidirectional laminar airflow, particularly in orthopedic surgery or patients warmed with a upper body warmer. ^[48]^ Furthermore, a previous study also confirmed that pathogenic organisms can be also be found in the hose of the forced-air warming system.^[49]^ Second, we only included patients with ASA grade I or II; however, patients with ASA grade III to IV have a higher risk for perioperative hypothermia.^[50]^ Third, we adopted 2 methods of measuring core temperature which may have affected the accuracy of results. However, the use of infrared tympanic thermometer is not invasive and may be more acceptable to conscious patients though the optimal temperature measurement method has not been determined. Finally, this was only a single-center randomized controlled study with limited sample size, more patients and multi-center prospective trials are needed to further verify the conclusion of this study.

## 5 Conclusion

In summary, prewarming patients for 30 minutes before the induction of anesthesia combined with a periprocedural forced-air warming system for patients undergoing VATS could improve perioperative core temperature and patients’ thermal comfort though albeit with no improvement in postoperative shivering and severity.

## Author contributions

**Conceptualization:** Yan Xiao, Chunmiao Hou, Huiying Xu.

**Data curation:** Yan Xiao.

**Formal analysis:** Na Lv.

**Investigation:** Na Lv.

**Methodology:** Na Lv.

**Project administration:** Chunmiao Hou.

**Resources:** Chunmiao Hou.

**Software:** Chunguang Ren.

**Supervision:** Chunguang Ren, Huiying Xu.

**Validation:** Rui Zhang, Huiying Xu.

**Writing – original draft:** Yan Xiao, Rui Zhang.

**Writing – review & editing:** Yan Xiao, Rui Zhang.
